# Supplementary material for: The Importance of Environmentally Acquired Bacterial Symbionts for the Squash Bug (Anasa tristis), a Significant Agricultural Pest
Source: Front Microbiol. 2021 Oct 4;12:719112. doi: 10.3389/fmicb.2021.719112 (PMC8521078; doi:10.3389/fmicb.2021.719112)
Supplement: Supplementary Tables and Figures — This file contains all supplementary figures and tables, except for Supplementary Table 5. [file Data_Sheet_1.docx]

**The importance of environmentally-acquired bacterial symbionts for the squash bug *Anasa tristis*, a significant agricultural pest**

Tarik S. Acevedo^1^, Gregory P. Fricker^1^, Justine R. Garcia^1,2^, Tiffanie Alcaide^1^, Aileen Berasategui^1^, Kayla S. Stoy^1^, Nicole M. Gerardo^1*^

^1^Department of Biology, Emory University, 1510 Clifton Road, Atlanta, GA, 30322, USA

^2^Department of Biology, New Mexico Highlands University, 1005 Diamond Ave, Las Vegas, NM, 87701, USA

***Correspondence:**

Nicole Gerardo

Nicole.gerardo@emory.edu

**Supplementary Figures and Tables.** This file contains all supplementary tables and figures except for Supplementary Table 5, which is provided in a standalone file.

**Supplementary Figure 1. Midgut of *Anasa tristis*.** ﻿(A) Alimentary tract dissected from a fourth instar nymph, with dashed box emphasizing the M4 crypt region. (B) GFP-labeled *Caballeronia* symbionts within M4 crypts. ﻿M1, midgut first section; M2, midgut second section; M3, midgut third section; M4B, M4 bulb; M4, midgut fourth section with crypts; H, hindgut. Photos by S. Mendiola and J. Chen.


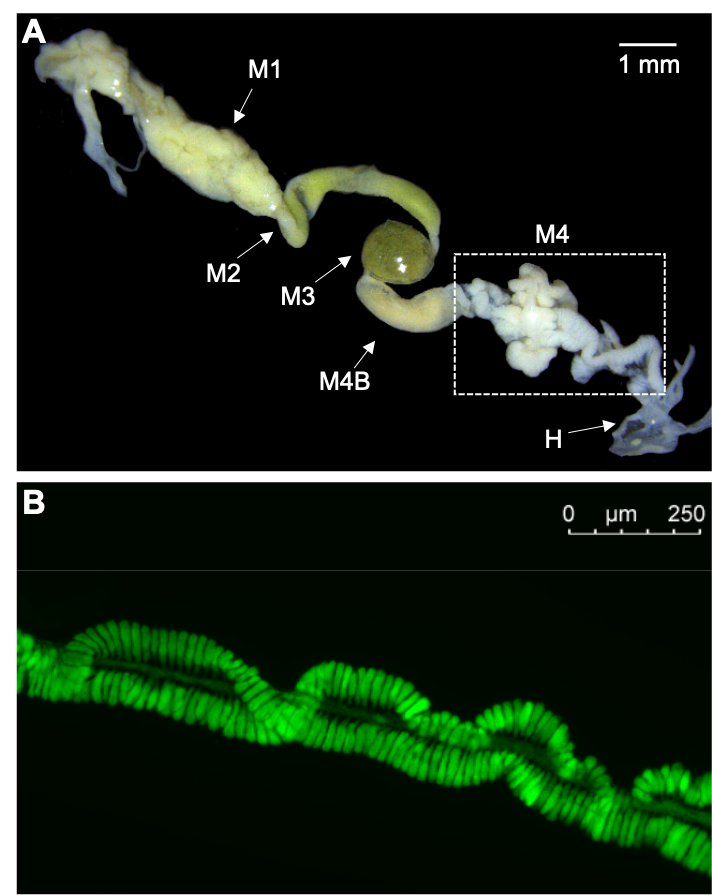


**Supplementary Table 1. Sampling depth and characteristics of processed high-throughput sequencing** **libraries**. See materials and methods for details.

| **Sample** | **No. Raw Reads** | **No. Processed Reads** | **No. *Caballeronia* Reads** | **% *Caballeronia* Reads** | **Accession Number** |
| --- | --- | --- | --- | --- | --- |
| SB33-m1 | 127,976 | 101,129 | 22,306 | 22.1 | SRR15264425 |
| SB34-m1 | 135,311 | 99,738 | 40,194 | 40.3 | SRR15264424 |
| SB35-m1 | 129,943 | 105,604 | 21,930 | 20.8 | SRR15264423 |
| SB36-m1 | 148,449 | 110,127 | 11,260 | 10.2 | SRR15264422 |
| SB11-m4 | 131,497 | 106,878 | 104,379 | 97.7 | SRR15264343 |
| SB33-m4 | 157,265 | 121,165 | 98,823 | 81.5 | SRR15264342 |
| SB34-m4 | 95,709 | 75,902 | 57,897 | 76.3 | SRR15264341 |
| SB35-m4 | 78,500 | 61,120 | 51,441 | 84.2 | SRR15264340 |
| SB36-m4 | 123,032 | 92,575 | 259 | 0.3 | SRR15264339 |
| SB74-wg | 89,147 | 70,057 | 47,417 | 67.7 | SRR15264421 |
| SB80-wg | 203,580 | 156,361 | 126,183 | 80.7 | SRR15264419 |
| SB84-wg | 138,314 | 105,484 | 38,849 | 36.8 | SRR15264418 |
| SB85-wg | 127,780 | 101,425 | 63,880 | 63.0 | SRR15264417 |
| SB86-wg | 142,729 | 109,106 | 4,168 | 3.8 | SRR15264416 |
| **Total:** | **1,829,232** | **1,416,671** | **688,986** | **--** | **--** |

**Supplementary Table 2**. Isolation of GFP-labeled bacteria SQ4a from eggs washed in solutions with alternative bacterial concentrations. CFUs indicates Colony Forming Units.

| **Estimated concentration of inoculation solution (CFUs/uL)** | **Total estimate of CFUs on sample of two eggs** | ***Caballeronia* detected?** |
| --- | --- | --- |
| 5.8 x 10^3^ | 97 | yes |
| 5.8 x 10^3^ | 219 | yes |
| 5.8 x 10^3^ | 580 | yes |
| 5.8 x 10^2^ | 5 | yes |
| 5.8 x 10^2^ | 14 | yes |
| 5.8 x 10^2^ | 3 | yes |
| 3.5 x 10^1^ | 1 | yes |
| 3.5 x 10^1^ | 17 | yes |
| 3.5 x 10^1^ | 2 | yes |
| 3.5 | 0 | no |
| 3.5 | 0 | no |
| 3.5 | 0 | no |

**Supplementary Table 3. *Caballeronia* spp. bacteria isolated from *Anasa tristis.*** Location abbreviations indicate: BCI - BCI Research Laboratory, Columbia, Missouri; CRY - Crystal Organic Farms, Newborn, Georgia; DEC - DeCamp Gardens, Albion, Indiana; FFA - Farlow Farm, Archdale, Indiana; FFF - Front Field Farm, Winterville, Georgia; MGA - Mission Garden, Tucson, Arizona; MLF - Merry Lea Farm, Albion, Indiana; NDG - North Dekalb Garden, Atlanta, Georgia; OAK - Oakhurst Community Garden, Decatur, Georgia; OXF - Oxford Farm, Oxford, Georgia; SBG - Stoy/Barse Gardens, Auburn, Indiana; TMF - Ten Mothers Farm, Hillsborough, North Carolina; UFL – University of Florida Garden, Gainesville, Florida; and WOG - Woodland Gardens, Atlanta, Georgia. All were collected within the United States. Classification and match to genus are based on the Ribosomal Database Project RDP classifier.

| **Isolate Name** | **Individual** | **Life Stage** | **Tissue** | **State** | **Site** | **NCBI** | **Family** | ***Genus*** | **Match to Genus** |
| --- | --- | --- | --- | --- | --- | --- | --- | --- | --- |
| 3_56_M4_b | 3.56 | 3 | M4 crypt | Georgia | CRY | KX239758 | Burkholderiaceae | *Caballeronia* | 100 |
| 3_56_M4_c | 3.56 | 3 | M4 crypt | Georgia | CRY | KX239759 | Burkholderiaceae | *Caballeronia* | 100 |
| 3_57_M4_a | 3.57 | 3 | M4 crypt | Georgia | CRY | KX239760 | Burkholderiaceae | *Caballeronia* | 100 |
| 3_57_M4_b | 3.57 | 3 | M4 crypt | Georgia | CRY | KX239761 | Burkholderiaceae | *Caballeronia* | 100 |
| 3_57_M4_c | 3.57 | 3 | M4 crypt | Georgia | CRY | KX239762 | Burkholderiaceae | *Caballeronia* | 100 |
| 4_50_M4_a | 4.5 | 4 | M4 crypt | Georgia | CRY | KX239751 | Burkholderiaceae | *Caballeronia* | 100 |
| 4_50_M4_c | 4.5 | 4 | M4 crypt | Georgia | CRY | KX239763 | Burkholderiaceae | *Caballeronia* | 100 |
| 4_51_M4_a | 4.51 | 4 | M4 crypt | Georgia | CRY | KX239764 | Burkholderiaceae | *Caballeronia* | 100 |
| 4_51_M4_b | 4.51 | 4 | M4 crypt | Georgia | CRY | KT259148 | Burkholderiaceae | *Caballeronia* | 100 |
| 4_51_M4_c | 4.51 | 4 | M4 crypt | Georgia | CRY | KT259151 | Burkholderiaceae | *Caballeronia* | 100 |
| 5_46_M4_a | 5.46 | 5 | M4 crypt | Georgia | CRY | KX239767 | Burkholderiaceae | *Caballeronia* | 100 |
| 5_46_M4_b | 5.46 | 5 | M4 crypt | Georgia | CRY | KX239768 | Burkholderiaceae | *Caballeronia* | 100 |
| A32_M4_a | A.32 | A | M4 crypt | Georgia | CRY | KX239752 | Burkholderiaceae | *Caballeronia* | 100 |
| A32_M4_b | A.32 | A | M4 crypt | Georgia | CRY | KX239753 | Burkholderiaceae | *Caballeronia* | 100 |
| A32_M4_c | A.32 | A | M4 crypt | Georgia | CRY | KX239754 | Burkholderiaceae | *Caballeronia* | 99 |
| A33_M4_a | A.33 | A | M4 crypt | Georgia | CRY | KX239755 | Burkholderiaceae | *Caballeronia* | 100 |
| A33_M4_c | A.33 | A | M4 crypt | Georgia | CRY | KX239756 | Burkholderiaceae | *Caballeronia* | 100 |
| ATUFL_F1_KS43 | ATUFL_F1 | A | M4 crypt | Florida | UFL | MZ264256 | Burkholderiaceae | *Caballeronia* | 100 |
| ATUFL_F1_KS4A | ATUFL_F1 | A | M4 crypt | Florida | UFL | MZ264265 | Burkholderiaceae | *Caballeronia* | 100 |
| ATUFL_F2_KS42 | ATUFL_F2 | A | M4 crypt | Florida | UFL | MZ264255 | Burkholderiaceae | *Caballeronia* | 100 |
| ATUFL_F2_KS9A | ATUFL_F2 | A | M4 crypt | Florida | UFL | MZ264267 | Burkholderiaceae | *Caballeronia* | 100 |
| ATUFL_F3_KS10A | ATUFL_F3 | A | M4 crypt | Florida | UFL | MZ264276 | Burkholderiaceae | *Caballeronia* | 100 |
| ATUFL_M1_KS5A | ATUFL_M1 | A | M4 crypt | Florida | UFL | MZ264275 | Burkholderiaceae | *Caballeronia* | 100 |
| ATUFL_M2_KS44 | ATUFL_M2 | A | M4 crypt | Florida | UFL | MZ264257 | Burkholderiaceae | *Caballeronia* | 100 |
| AZ1_KS37 | AZ1 | A | M4 crypt | Arizona | MGA | MZ264251 | Burkholderiaceae | *Caballeronia* | 100 |
| AZ10_KS36 | AZ10 | A | M4 crypt | Arizona | MGA | MZ264250 | Burkholderiaceae | *Caballeronia* | 100 |
| AZ7_KS35 | AZ7 | A | M4 crypt | Arizona | MGA | MZ264272 | Burkholderiaceae | *Caballeronia* | 100 |
| GACF3 | GACF3 | A | M4 crypt | Gerogia | CRY | MZ264239 | Burkholderiaceae | *Caballeronia* | 100 |
| GACF4 | GACF4 | A | M4 crypt | Gerogia | CRY | MZ264273 | Burkholderiaceae | *Caballeronia* | 100 |
| GACF5 | GACF5 | A | M4 crypt | Georgia | CRY | MZ264240 | Burkholderiaceae | *Caballeronia* | 100 |
| GAFFF1 | GAFFF1 | A | M4 crypt | Georgia | FFF | MZ264232 | Burkholderiaceae | *Caballeronia* | 100 |
| GAFFF2 | GAFFF2 | A | M4 crypt | Georgia | FFF | MZ264233 | Burkholderiaceae | *Caballeronia* | 100 |
| GAFFF3 | GAFFF3 | A | M4 crypt | Georgia | FFF | MZ264269 | Burkholderiaceae | *Caballeronia* | 100 |
| GAFFF5 | GAFFF5 | A | M4 crypt | Georgia | FFF | MZ264234 | Burkholderiaceae | *Caballeronia* | 100 |
| GAOx1 | GAOx1 | A | M4 crypt | Georgia | OXF | MZ264271 | Burkholderiaceae | *Caballeronia* | 100 |
| GAWG1_1 | GAWG1_1 | A | M4 crypt | Georgia | WOG | MZ264235 | Burkholderiaceae | *Caballeronia* | 100 |
| GAWG2_1 | GAWG2_1 | A | M4 crypt | Georgia | WOG | MZ264237 | Burkholderiaceae | *Caballeronia* | 100 |
| GAWG2_2 | GAWG2_2 | A | M4 crypt | Georgia | WOG | MZ264238 | Burkholderiaceae | *Caballeronia* | 100 |
| GAWG2_4 | GAWG2_4 | A | M4 crypt | Georgia | WOG | MZ264274 | Burkholderiaceae | *Caballeronia* | 100 |
| INDec2 | INDec2 | A | M4 crypt | Indiana | DEC | MZ264241 | Burkholderiaceae | *Caballeronia* | 100 |
| INML1 | INML1 | A | M4 crypt | Indiana | MLF | MZ264270 | Burkholderiaceae | *Caballeronia* | 100 |
| INML2 | INML2 | A | M4 crypt | Indiana | MLF | MZ264242 | Burkholderiaceae | *Caballeronia* | 100 |
| INML3_ML3B | INML3 | A | M4 crypt | Indiana | MLF | MZ264243 | Burkholderiaceae | *Caballeronia* | 100 |
| INML3_ML3Y | INML3 | A | M4 crypt | Indiana | MLF | MZ264246 | Burkholderiaceae | *Caballeronia* | 100 |
| INML5 | INML5 | A | M4 crypt | Indiana | MLF | MZ264244 | Burkholderiaceae | *Caballeronia* | 100 |
| INSB1 | INSB1 | A | M4 crypt | Indiana | SBG | MZ264245 | Burkholderiaceae | *Caballeronia* | 100 |
| NCF2_F2 | NCF2 | A | M4 crypt | North Carolina | FFA | MZ264248 | Burkholderiaceae | *Caballeronia* | 100 |
| NCF4 | NCF4 | A | M4 crypt | North Carolina | FFA | MZ264268 | Burkholderiaceae | *Caballeronia* | 100 |
| NCTM1 | NCTM1 | A | M4 crypt | North Carolina | TMF | MZ264247 | Burkholderiaceae | *Caballeronia* | 100 |
| NCTM5 | NCTM5 | A | M4 crypt | North Carolina | TMF | MZ264249 | Burkholderiaceae | *Caballeronia* | 100 |
| SB13_M4_a | SB13 | A | M4 crypt | Georgia | WOG | KT259185 | Burkholderiaceae | *Caballeronia* | 100 |
| SB14_M4_a | SB14 | A | M4 crypt | Georgia | WOG | KT259132 | Burkholderiaceae | *Caballeronia* | 100 |
| SB17_M4_b | SB17 | A | M4 crypt | Georgia | OAK | MH636870 | Burkholderiaceae | *Caballeronia* | 100 |
| SB18_M4_b | SB18 | A | M4 crypt | Georgia | OAK | KT259186 | Burkholderiaceae | *Caballeronia* | 100 |
| SB19_M4_a | SB19 | A | M4 crypt | Georgia | OAK | KT259133 | Burkholderiaceae | *Caballeronia* | 100 |
| SB19_M4_b | SB19 | A | M4 crypt | Georgia | OAK | KT259134 | Burkholderiaceae | *Caballeronia* | 100 |
| SB1b_LB | SB1 | A | M4 crypt | Georgia | CRY | KT259135 | Burkholderiaceae | *Caballeronia* | 100 |
| SB1B_V | SB1 | A | M4 crypt | Georgia | CRY | KT259136 | Burkholderiaceae | *Caballeronia* | 100 |
| SB1c_LB | SB1 | A | M4 crypt | Georgia | CRY | KT259137 | Burkholderiaceae | *Caballeronia* | 100 |
| SB1c_V | SB1 | A | M4 crypt | Georgia | CRY | KT259138 | Burkholderiaceae | *Caballeronia* | 100 |
| SB1d_LB | SB1 | A | M4 crypt | Georgia | CRY | KT259139 | Burkholderiaceae | *Caballeronia* | 100 |
| SB1e_LB | SB1 | A | M4 crypt | Georgia | CRY | KT259140 | Burkholderiaceae | *Caballeronia* | 100 |
| SB1e_V | SB1 | A | M4 crypt | Georgia | CRY | KT259141 | Burkholderiaceae | *Caballeronia* | 100 |
| SB1f_LB | SB1 | A | M4 crypt | Georgia | CRY | KT259142 | Burkholderiaceae | *Caballeronia* | 100 |
| SB1f_V | SB1 | A | M4 crypt | Georgia | CRY | KT259143 | Burkholderiaceae | *Caballeronia* | 100 |
| SB1g_LB | SB1 | A | M4 crypt | Georgia | CRY | KT259144 | Burkholderiaceae | *Caballeronia* | 100 |
| SB1g_V | SB1 | A | M4 crypt | Georgia | CRY | KT259145 | Burkholderiaceae | *Caballeronia* | 100 |
| SB1h_LB | SB1 | A | M4 crypt | Georgia | CRY | KT259146 | Burkholderiaceae | *Caballeronia* | 100 |
| SB22_M4_a | SB22 | A | M4 crypt | Georgia | CRY | KT259147 | Burkholderiaceae | *Caballeronia* | 100 |
| SB22_M4_b | SB22 | A | M4 crypt | Georgia | CRY | KT259187 | Burkholderiaceae | *Caballeronia* | 100 |
| SB23_M4_c | SB23 | A | M4 crypt | Georgia | CRY | KT259183 | Burkholderiaceae | *Caballeronia* | 100 |
| SB23_M4_d | SB23 | A | M4 crypt | Georgia | CRY | KT259188 | Burkholderiaceae | *Caballeronia* | 100 |
| SB24_M4_a | SB24 | A | M4 crypt | Georgia | CRY | KT259148 | Burkholderiaceae | *Caballeronia* | 100 |
| SB25_M4_b | SB25 | A | M4 crypt | Georgia | CRY | KT259149 | Burkholderiaceae | *Caballeronia* | 100 |
| SB25_M4_c | SB25 | A | M4 crypt | Georgia | CRY | KT259150 | Burkholderiaceae | *Caballeronia* | 100 |
| SB26_M4_a | SB26 | A | M4 crypt | Georgia | CRY | KT259189 | Burkholderiaceae | *Caballeronia* | 100 |
| SB26_M4_b | SB26 | A | M4 crypt | Georgia | CRY | MH636872 | Burkholderiaceae | *Caballeronia* | 100 |
| SB27_M4_a | SB27 | A | M4 crypt | Georgia | CRY | KT259184 | Burkholderiaceae | *Caballeronia* | 100 |
| SB28_M4_a | SB28 | A | M4 crypt | Missouri | BCI | MH791154 | Burkholderiaceae | *Caballeronia* | 100 |
| SB29_M4_b | SB29 | A | M4 crypt | Missouri | BCI | KT259190 | Burkholderiaceae | *Caballeronia* | 100 |
| SB29_M4_c | SB29 | A | M4 crypt | Missouri | BCI | KT259151 | Burkholderiaceae | *Caballeronia* | 100 |
| SB30_M4_a | SB30 | A | M4 crypt | Missouri | BCI | KT259191 | Burkholderiaceae | *Caballeronia* | 100 |
| SB31_M4_b | SB31 | A | M4 crypt | Missouri | BCI | MH636871 | Burkholderiaceae | *Caballeronia* | 100 |
| SB7b | SB7 | A | M4 crypt | Georgia | NDG | KT259152 | Burkholderiaceae | *Caballeronia* | 100 |
| SB7d | SB7 | A | M4 crypt | Georgia | NDG | KT259153 | Burkholderiaceae | *Caballeronia* | 100 |
| SB7f | SB7 | A | M4 crypt | Georgia | NDG | KT259154 | Burkholderiaceae | *Caballeronia* | 100 |
| SB7g | SB7 | A | M4 crypt | Georgia | NDG | KT259155 | Burkholderiaceae | *Caballeronia* | 100 |
| SB7h | SB7 | A | M4 crypt | Georgia | NDG | KT259156 | Burkholderiaceae | *Caballeronia* | 100 |
| SB8b | SB8 | A | M4 crypt | Georgia | NDG | KT259157 | Burkholderiaceae | *Caballeronia* | 100 |
| SB8c | SB8 | A | M4 crypt | Georgia | NDG | KT259158 | Burkholderiaceae | *Caballeronia* | 100 |
| SB8d | SB8 | A | M4 crypt | Georgia | NDG | KT259159 | Burkholderiaceae | *Caballeronia* | 100 |
| SB8e | SB8 | A | M4 crypt | Georgia | NDG | KT259160 | Burkholderiaceae | *Caballeronia* | 100 |
| SB8f | SB8 | A | M4 crypt | Georgia | NDG | KT259161 | Burkholderiaceae | *Caballeronia* | 100 |
| SB8g | SB8 | A | M4 crypt | Georgia | NDG | KT259162 | Burkholderiaceae | *Caballeronia* | 100 |
| SQ4a | SQ4 | A | M4 crypt | Georgia | OAK | MH636869 | Burkholderiaceae | *Caballeronia* | 99 |
| SQ4f | SQ4 | A | M4 crypt | Georgia | OAK | KT259164 | Burkholderiaceae | *Caballeronia* | 93 |
| SQ5a | SQ5 | A | M4 crypt | Georgia | OAK | KT259166 | Burkholderiaceae | *Caballeronia* | 100 |
| SQ5b | SQ5 | A | M4 crypt | Georgia | OAK | KT259167 | Burkholderiaceae | *Caballeronia* | 100 |
| SQ5c | SQ5 | A | M4 crypt | Georgia | OAK | KT259168 | Burkholderiaceae | *Caballeronia* | 100 |
| SQ5d | SQ5 | A | M4 crypt | Georgia | OAK | KT259169 | Burkholderiaceae | *Caballeronia* | 100 |
| SQ5e | SQ5 | A | M4 crypt | Georgia | OAK | KT259170 | Burkholderiaceae | *Caballeronia* | 100 |
| SQ5f | SQ5 | A | M4 crypt | Georgia | OAK | KT259171 | Burkholderiaceae | *Caballeronia* | 100 |
| SQ5g | SQ5 | A | M4 crypt | Georgia | OAK | KT259172 | Burkholderiaceae | *Caballeronia* | 100 |
| SQ5h | SQ5 | A | M4 crypt | Georgia | OAK | KT259173 | Burkholderiaceae | *Caballeronia* | 100 |
| SQ5i | SQ5 | A | M4 crypt | Georgia | OAK | KT259174 | Burkholderiaceae | *Caballeronia* | 100 |
| SQ6a | SQ6 | A | M4 crypt | Georgia | OAK | KT259175 | Burkholderiaceae | *Caballeronia* | 100 |
| SQ6b | SQ6 | A | M4 crypt | Georgia | OAK | KT259176 | Burkholderiaceae | *Caballeronia* | 100 |
| SQ6d | SQ6 | A | M4 crypt | Georgia | OAK | KT259177 | Burkholderiaceae | *Caballeronia* | 100 |
| SQ6e | SQ6 | A | M4 crypt | Georgia | OAK | KT259178 | Burkholderiaceae | *Caballeronia* | 100 |
| SQ6f | SQ6 | A | M4 crypt | Georgia | OAK | KT259179 | Burkholderiaceae | *Caballeronia* | 100 |
| SQ6g | SQ6 | A | M4 crypt | Georgia | OAK | KT259180 | Burkholderiaceae | *Caballeronia* | 100 |
| SQ6h | SQ6 | A | M4 crypt | Georgia | OAK | KT259181 | Burkholderiaceae | *Caballeronia* | 100 |
| SQ6i | SQ6 | A | M4 crypt | Georgia | OAK | KT259182 | Burkholderiaceae | *Caballeronia* | 100 |
| WG_1_5s_s | WG_1_5 | A | M4 crypt | Georgia | WOG | MZ264236 | Burkholderiaceae | *Caballeronia* | 100 |

**Supplementary Table 4. Non-*Caballeronia* spp. bacteria isolated from *Anasa tristis.*** Abbreviations are as in Supplementary Table 3. Classification and match to genus are based on the Ribosomal Database Project RDP classifier.

| **Isolate**  **Name** | **Individual** | **Location** | **Life**  **Stage** | **Tissue** | **NCBI**  **Accession** | **Phylum** | **Class** | **Order** | **Family** | **Genus** | **Match**  **to Genus** |
| --- | --- | --- | --- | --- | --- | --- | --- | --- | --- | --- | --- |
| SB21_M4_a | SB21 | CRY | A | M4 crypt | MH828198 | Firmicutes | Bacilli | Bacillales | Bacillaceae 1 | *Bacillus* | 100 |
| SB23_M4_a | SB23 | CRY | A | M4 crypt | MH828199 | Firmicutes | Bacilli | Bacillales | Bacillaceae 1 | *Bacillus* | 100 |
| SB27_M4_b | SB27 | CRY | A | M4 crypt | MH828202 | Firmicutes | Bacilli | Bacillales | Bacillaceae 1 | *Bacillus* | 100 |
| SB28_M4_c | SB28 | BCI | A | M4 crypt | MH828205 | Firmicutes | Bacilli | Bacillales | Bacillaceae 1 | *Bacillus* | 100 |
| SB19_M4_c | SB19 | OAK | A | M4 crypt | MH828197 | Firmicutes | Bacilli | Bacillales | Paenibacillaceae 1 | *Paenibacillus* | 100 |
| SB27_M4_c | SB27 | CRY | A | M4 crypt | MH828203 | Firmicutes | Bacilli | Bacillales | Staphylococcaceae | *Staphylococcus* | 100 |
| SB23_M4_b | SB23 | CRY | A | M4 crypt | MH828200 | Firmicutes | Bacilli | Lactobacillales | Enterococcaceae | *Enterococcus* | 100 |
| A33_M4_g | A.33 | CRY | A | M4 crypt | MH828196 | Proteobacteria | Gammaproteobacteria | Enterobacteriales | Enterobacteriaceae | *Klebsiella* | 93 |
| SB31_M4_a | SB31 | BCI | A | M4 crypt | MH828207 | Proteobacteria | Gammaproteobacteria | Enterobacteriales | Enterobacteriaceae | *Klebsiella* | 94 |
| 2_61_a | 2.61 | CRY | 2 | Whole Body | MH828191 | Proteobacteria | Gammaproteobacteria | Enterobacteriales | Enterobacteriaceae | *Serratia* | 100 |
| 2_61_b | 2.61 | CRY | 2 | Whole Body | MH828192 | Proteobacteria | Gammaproteobacteria | Enterobacteriales | Enterobacteriaceae | *Serratia* | 100 |
| A33_M4_b | A.33 | CRY | A | M4 crypt | MH828193 | Proteobacteria | Gammaproteobacteria | Pseudomonadales | Moraxellaceae | *Acinetobacter* | 100 |
| SB28_M4_b | SB28 | BCI | A | M4 crypt | MH828204 | Proteobacteria | Gammaproteobacteria | Pseudomonadales | Pseudomonadaceae | *Pseudomonas* | 100 |
| A33_M4_e | A.33 | CRY | A | M4 crypt | MH828195 | Proteobacteria | Gammaproteobacteria | Xanthomonadales | Xanthomonadaceae | *Stenotrophomonas* | 100 |

**Supplementary Figure 2.** **16S rRNA phylogeny of *Caballeronia* isolates from squash bug crypts, including symbionts of other true bugs species and other Burkholderiaceae.** Topology is the same as in Figure 2 in the main text. NCBI accession numbers for *A. tristis* symbionts are in Supplementary Table 2. All other accession numbers follow taxa names. Shaded boxes indicate isolates used in fitness assays.


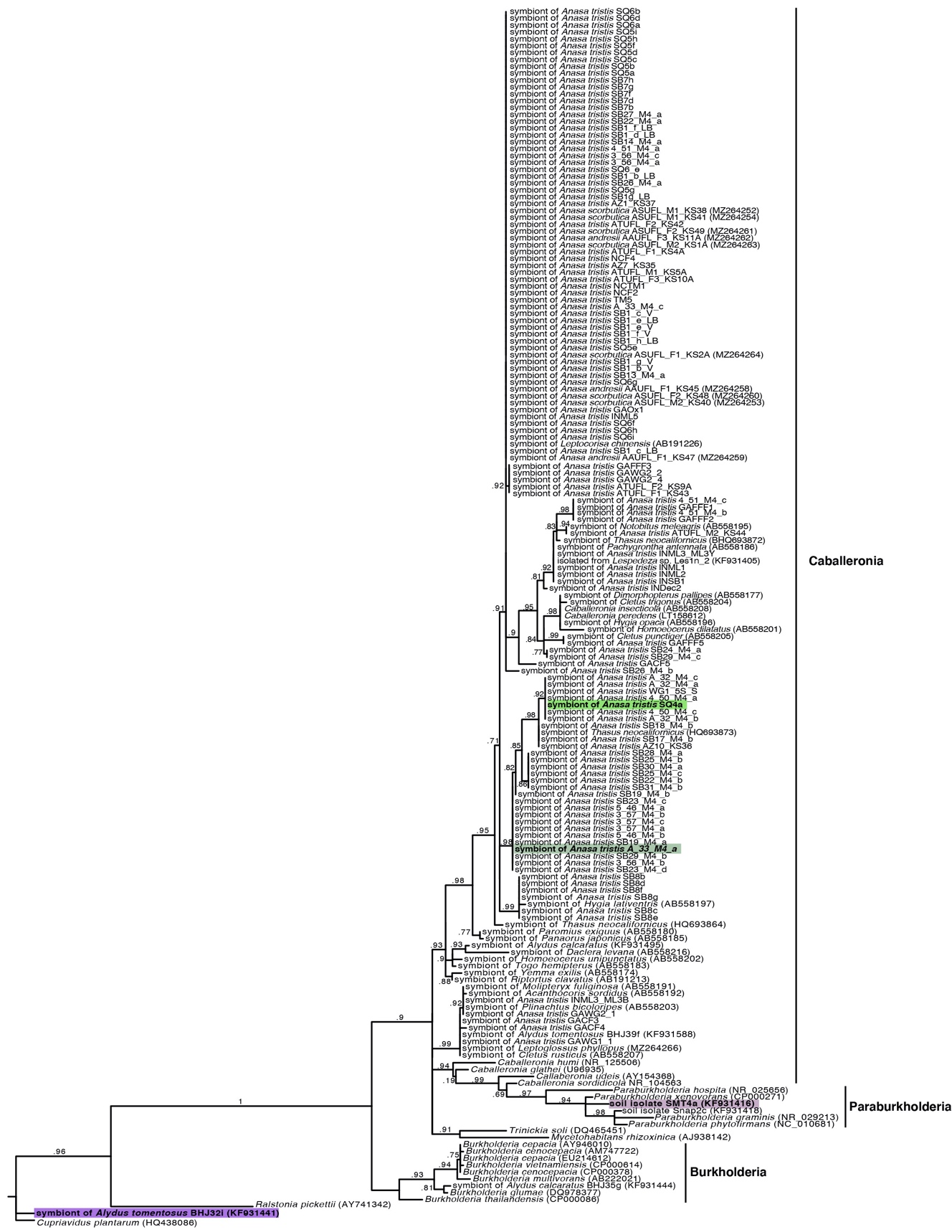


**Supplementary Figure 3. Pairwise sequence similarity of all 16s rRNA sequences included in Supplementary Figure 2.** Colors indicate proportion of bases that are identical. Taxa are ordered as in Supplementary Figure 2. Many of the *Anasa* spp. symbionts fall into one of six sequence groups (demarcated with dashed lines) with greater than 99% sequence identity that cluster together on the phylogeny. Most others do no show as clear of a pattern of high sequence similarity but fall on the phylogeny between Group 1 and Group 2.

­­

**Supplementary Table 6. Assessment of potential *Caballeronia* co-infection.** Of the 20 individuals from which multiple *Caballeronia* colonies were isolated, six individuals (rows in green) were detected to have been co-infected with multiple *Caballeronia* strains based on the bacteria from the individual falling in different parts of the phylogeny (Supplementary Figure 2) and in different sequence groups (Supplementary Figure 3). Sequence groups (Supplementary Figure 3) are listed in parentheses after each isolate name. NG refers to those bacteria that did not fall into one of the easily distinguishable sequence groups but that did fall within a well-supported clade within the phylogeny that does not contain any of the other sequence groups; these strains are between Group 1 and Group 2 in Supplementary Figure 3.

| **Individual** | **Isolates**  **(sequence group in parentheses)** | **Evidence for**  ***Caballeronia* co-infection** |
| --- | --- | --- |
| 3.56 | 3_56_M4_a (1), 3_56_M4_b (4), 3_56_M4_c (1) | yes |
| 3.57 | 3_57_M4_a (4), 3_57_M4_b (4), 3_57_M4_c (4) | no |
| 4.5 | 4_50_M4_a (2), 4_50_M4_c (2) | no |
| 4.51 | 4_51_M4_a (1), 4_51_M4_b (NG), 4_51_M4_c (NG) | yes |
| 5.46 | 5_46_M4_a (4), 5_46_M4_b (4) | no |
| A.32 | A32_M4_a (2), A32_M4_b (2), A32_M4_c (2) | no |
| A.33 | A33_M4_a (4), A33_M4_c (1) | yes |
| ATUFL_F1 | ATUFL_F1_KS43 (1), ATUFL_F1_KS4A (1) | no |
| ATUFL_F2 | ATUFL_F2_KS42 (1), ATUFL_F2_KS9A (1) | no |
| INML3 | INML3_ML3B (6), INML3_ML3Y (NG) | yes |
| SB19 | SB19_M4_a (4), SB19_M4_b (4) | no |
| SB1 | SB1b_LB (1), SB1B_V (1), SB1c_LB (1), SB1c_V (1), SB1d_LB (1), SB1e_LB (1), SB1e_V (1), SB1f_LB (1), SB1f_V (1), SB1g_LB (1), SB1g_V (1), SB1h_LB (1) | no |
| SB22 | SB22_M4_a (1), SB22_M4_b (3) | yes |
| SB23 | SB23_M4_c (4), SB23_M4_d (4) | no |
| SB25 | SB25_M4_b (3), SB25_M4_c (3) | no |
| SB26 | SB26_M4_a (1), SB26_M4_b (NG) | yes |
| SB7 | SB7b (1), SB7d (1), SB7d (1), SB7g (1), SB7h (1) | no |
| SB8 | SB8b (5), SB8c (5), SB8d (5), SB8e (5), SB8f (5), SB8g (5) | no |
| SQ5 | SQ5a (1), SQ5b (1), SQ5c (1), SQ5d (1), SQ5e (1), SQ5f (1), SQ5g (1), SQ5h (1), SQ5i (1) | no |
| SQ6 | SQ6a (1), SQ6b (1), SQ6d (1), SQ6e (1), SQ6f (1), SQ6g (1), SQ6h (1), SQ6i (1) | no |

**Supplementary Figure 4. Set up for rearing squash bugs on plants.** Squash plants are grown from sterilized seeds in pots that extend into a plastic bin filled with water. The water is fertilized with Botanicare® Pure Blend Pro Grow Organic Fertilizer. The bin is filled with fertilized water until reaching the bottom of the pot.


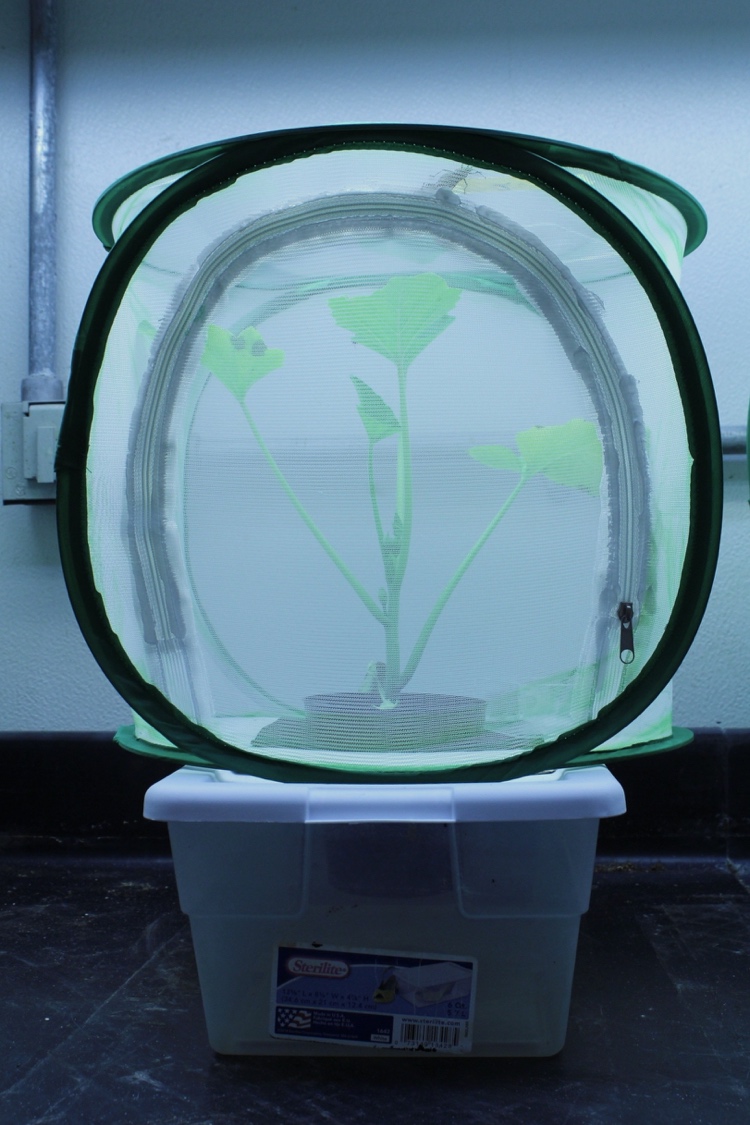


**Supplementary Figure 5. Estimated population size of *Caballeronia* SQ4a in adult female and male crypts.** Insects were reared on plants. Bars indicate means, and points indicate estimate for each individual.

**Supplementary Table 7.** Results of Tukey’s post-hoc comparisons of development time in days from hatch to each instar when fed alternative bacterial strains. Each cell contains the z-value. *indicates adjusted *P* < 0.05, ** adjusted *P* < 0.01, *** adjusted *P* < 0.001. All significant differences are highlighted in bold.

| **Comparison** | **Hatch to 3^rd^ Instar** | **Hatch to 4^th^ Instar** | **Hatch to 5^th^ Instar** | **Hatch to Adult** |
| --- | --- | --- | --- | --- |
| H_2_O - SQ4a | **3.18*** | **18.32***** | **27.01***** | **21.96***** |
| H_2_O - A33 | **3.36**** | **17.43***** | **25.05***** | **19.36***** |
| H_2_O - SMT4a | 0.13 | 0.50 | **6.61***** | **5.80***** |
| H_2_O - BHJ | 1.00 | -1.73 | 0.37 | -1.57 |
| SQ4a - A33 | 0.35 | 0.06 | -0.54 | -1.72 |
| SQ4a - SMT4a | **-2.88*** | **-17.70***** | **-19.06***** | **-14.76***** |
| SQ4a - BHJ | -2.44 | **-19.90***** | **-26.30***** | **-23.25***** |
| A33 - SMT4a | **-3.07*** | **-16.94***** | **-17.68***** | **-12.64***** |
| A33 - BHJ | -2.67 | **-18.92***** | **-24.42***** | **-20.65***** |
| SMT4a - BHJ | 0.57 | -1.10 | **-6.17***** | **-7.19***** |

**Supplementary Table 8.** Results of Tukey’s post-hoc comparisons of impact of bacterial inoculation treatment on adult pronotal width. Each cell contains the z-value. *indicates adjusted *P* < 0.05, ** adjusted *P* < 0.01, *** adjusted *P* < 0.001. All significant differences are highlighted in bold.

| **Comparison** | **Difference in Means**  **log-transformed pronotal width, mm** |
| --- | --- |
| H_2_O – SQ4a | **-0.06***** |
| H_2_O - A33 | **-0.06***** |
| H_2_O – SMT4a | -0.02 |
| H_2_O – BHJ | -0.01 |
| SQ4a - A33 | 0.002 |
| SQ4a – SMT4a | **0.04***** |
| SQ4a – BHJ | **0.05***** |
| A33 – SMT4a | **0.04***** |
| A33 – BHJ | **0.05***** |
| SMT4a – BHJ | 0.01 |
